# Impaired Recovery Sleep in Individuals with Alzheimer’s Disease – The RESTED Study

**DOI:** 10.1002/alz.092206

**Published:** 2025-01-03

**Authors:** Jonathan Blackman, Victoria Grace Gabb, Hamish Morrison, Nicholas Turner, Bijetri Biswas, Haoxuan Li, Alan Whone, Elizabeth Coulthard

**Affiliations:** ^1^ North Bristol NHS Trust, Southmead Hospital, Bristol United Kingdom; ^2^ University of Bristol, Bristol United Kingdom; ^3^ North Bristol NHS Trust, Bristol United Kingdom

## Abstract

**Background:**

Homeostatic sleep regulation is theorized to be governed by the ‘Two Process Model’ where circadian rhythm (process C) and homeostatic sleep pressure (process S) interact to determine sleep versus wakefulness. Sleep pressure accumulated during prolonged wakefulness increases the duration and intensity of subsequent ‘recovery’ sleep. Multiple sleep abnormalities are associated with Alzheimer’s Disease (AD) Dementia and Lewy Body Dementia (LBD). This study seeks to determine whether recovery sleep duration may also be compromised.

**Method:**

RESTED (Remote Evaluation of Sleep To enhance understanding in Early Dementia) was an observational cohort study with three subgroups: AD MCI/dementia, LBD MCI/dementia, and healthy controls (HC). Participants wore a wrist actigraph for 55 nights continuously and provided daily sleep diaries. Recovery sleep was assessed using a random intercept lagged panel model with a dependent variable of total sleep time (TST). Prespecified covariates included age, sex, Geriatric Depression Score, Generalised Anxiety Disorder 7‐Score and participant subgroup (AD/LBD/HC). Compensatory sleep represented a ‘within‐person’ carry‐over effect modelled with an autoregressive parameter (alpha). A negative value implies low TST nights are typically followed by higher TST nights, indicating compensatory or recovery sleep Interaction terms determined if alpha was affected by participant group or age.

**Result:**

40 participants (AD = 10, LBD = 10, HC = 20) underwent full study procedures. Mean (SD) age was 70.9 (5.9) [AD = 69.2(7.9), LBD = 73.9(2.8), HC = 70.2(5.7)]. 9/40 (22.5%) participants were female (AD = 2/10, LB = 2/10, HC = 5/20). Mean (SD) Montreal Cognitive Assessment Score was 24.8 (4) [AD = 23.0(5), LBD = 21.9(4), HC = 27.1(1)].

Age (β = ‐0.11, SE = 0.05, p = 0.036), diagnosis of AD (β = ‐1.75, SE = 0.57, p = 0.004) and alpha (β = ‐0.94, SE = 0.31, p = 0.002) were all negatively associated with sleep duration. There were significant positive interactions between alpha and diagnosis of AD (β = 0.13, SE = 0.06, p = 0.026) and alpha and increasing age (β = 0.01, SE = 0.004, p = 0.001). No statistically significant effects were found linking LBD to sleep duration.

**Conclusion:**

Low TST nights were typically followed by higher TST nights suggesting that compensatory mechanisms were present. However, this relationship was attenuated both by the presence of AD and increasing age (approximately 10% per decade). Further research is needed to confirm these findings in larger cohorts and determine underlying pathophysiological mechanisms by which sleep recovery is compromised.

